# CdSe nanorod/TiO_2_ nanoparticle heterojunctions with enhanced solar- and visible-light photocatalytic activity

**DOI:** 10.3762/bjnano.8.273

**Published:** 2017-12-19

**Authors:** Fakher Laatar, Hatem Moussa, Halima Alem, Lavinia Balan, Emilien Girot, Ghouti Medjahdi, Hatem Ezzaouia, Raphaël Schneider

**Affiliations:** 1 Laboratory of Semiconductors, Nanostructures and Advanced Technology (LSNTA), Center for Research and Technology Energy, Tourist Route Soliman, BP 95, 2050 Hammam-Lif, Tunisia; 2 rue Grandville 54001 Nancy, France; 3 Laboratoire Réactions et Génie des Procédés (LRGP), UMR 7274 CNRS Université de Lorraine; 4 Institut Jean Lamour (IJL), UMR 7198 CNRS Université de Lorraine, BP 70239, 54506 Vandoeuvre-lès-Nancy Cedex, France,; 5 Institut de Science des Matériaux de Mulhouse (IS2M), CNRS UMR 7361, 15 rue Jean Starcky, 68093 Mulhouse, France

**Keywords:** CdSe nanorods, heterojunction, photocatalysis, TiO_2_

## Abstract

CdSe nanorods (NRs) with an average length of ≈120 nm were prepared by a solvothermal process and associated to TiO_2_ nanoparticles (Aeroxide^®^ P25) by annealing at 300 °C for 1 h. The content of CdSe NRs in CdSe/TiO_2_ composites was varied from 0.5 to 5 wt %. The CdSe/TiO_2_ heterostructured materials were characterized by XRD, TEM, SEM, XPS, UV–visible spectroscopy and Raman spectroscopy. TEM images and XRD patterns show that CdSe NRs with wurtzite structure are associated to TiO_2_ particles. The UV–visible spectra demonstrate that the narrow bandgap of CdSe NRs serves to increase the photoresponse of CdSe/TiO_2_ composites until ≈725 nm. The CdSe (2 wt %)/TiO_2_ composite exhibits the highest photocatalytic activity for the degradation of rhodamine B in aqueous solution under simulated sunlight or visible light irradiation. The enhancement in photocatalytic activity likely originates from CdSe sensitization of TiO_2_ and the heterojunction between these materials which facilitates electron transfer from CdSe to TiO_2_. Due to its high stability (up to ten reuses without any significant loss in activity), the CdSe/TiO_2_ heterostructured catalysts show high potential for real water decontamination.

## Introduction

The development of efficient photocatalysts to address environmental and energy needs, such as degradation of harmful organic compounds in water and in the air or the conversion of solar energy to chemical energy, for example, via water splitting to produce hydrogen, is the topic of numerous current research projects. Titanium dioxide (TiO_2_) has been widely investigated over the last three decades and has been demonstrated to be of high potential [1–2]. However, TiO_2_ suffers from two main drawbacks. First, due to its wide bandgap (*E*_g_ = 3.2 and 3.0 eV for anatase and rutile, respectively), TiO_2_ can only be activated by light with a wavelength of less than 390 nm to trigger the electron–hole separation. Second, TiO_2_ exhibits a low quantum efficiency due to the fast recombination of photogenerated charge carriers (electrons and holes).

To address these problems, a number of studies have been devoted to the improvement of light absorption and charge separation by hybridizing TiO_2_ with narrow bandgap semiconductors, doping with metal or nonmetal elements, association with noble metal particles, or constructing heterojunctions between TiO_2_ and graphene-based materials or carbon nitride (C_3_N_4_), acting as electron-transport materials [3–5]. All of these strategies have the common goal of decreasing the charge carrier recombination rate by increasing the spatial charge separation.

The interfacial electron transfer between two semiconductors has gained significant interest because the heterojunction improves both the optical absorption in the visible range and the charge separation yield and thus the charge carrier lifetime [3–8]. The photocatalytic activity is enhanced because oxidation of water by holes and reduction of oxygen by electrons are retained at two different sites.

Recently, many groups demonstrated that the creation of a heterojunction between TiO_2_ and CdS, CdSe or alloyed CdSeS nanoparticles allows the optical absorption of TiO_2_ to be extended into the visible light range and increases the photoconversion efficiency by improving the charge transfer [9–37]. CdSe is one of the most commonly used semiconductors due to its narrow bandgap (1.7 eV) and its band energies are located at relatively low potential. Despite the large number of reports describing the sensitization of TiO_2_ (used in the form of spherical particles, films, wires, tubes, etc.) with CdSe nanocrystals (generally quantum dots or spherical nanoclusters) [9–37], very little attention has been devoted to the influence of CdSe crystal morphology on the photocatalytic activity of the CdSe/TiO_2_ heterostructured photocatalysts. CdSe nanorods (NRs) and wires are of high interest for use as sensitizers for photocatalytic applications due to their high surface area, higher optical absorption cross section (as compared to spherical particles) and easier charge carrier separation [38]. Only a couple of reports describe the association of CdSe NRs with TiO_2_. Luo et al. used chemical vapor deposition to associate CdS, CdSe or CdSeS rods to TiO_2_ NRs arrays and demonstrated that the CdSeS/TiO_2_ heterostructure exhibits the highest performances as photoelectrode [39]. More recently, small CdSe NRs [40] or type II CdSe/CdSe*_x_*Te_1−_*_x_* NRs [41] were also used as light harvesters to develop TiO_2_-based solar cells. Core/shell TiO_2_/CdSe NRs were also prepared by growing CdSe quantum dots onto TiO_2_ NRs at high temperature [42].

In this paper, we report an investigation on the synthesis of CdSe-NR-sensitized TiO_2_ nanoparticles and on the use of these materials for the degradation of rhodamine B (RhB) in aqueous solution. Our results demonstrate that the composite containing 2 wt % CdSe NRs exhibits the optimal photocatalytic activity under solar or visible light irradiation. Moreover, the photocatalytic response was maintained after ten cycles in simulated sunlight. A possible mechanism is also discussed.

## Experimental

### Chemicals

Cadmium acetate (Cd(OAc), 99.995%, Sigma), sodium selenite (Na_2_SeO_3_, 99%, Sigma), diethylenetriamine (99%, Sigma), TiO_2_ nanoparticles (Sigma-Aldrich, Aeroxide^®^ P25, CAS: 13463-67-7), rhodamine B (RhB, >95%, Sigma), *tert*-butanol (*t*-BuOH, >99.5%, Sigma), *p*-benzoquinone (>98%, Sigma) and ethanol (>99%, anhydrous) were used as received without additional purification. All solutions were prepared using Milli-Q water (18.2 MΩ·cm, Millipore) as the solvent.

### Synthesis of CdSe nanorods

CdSe NRs were synthetized via a solvothermal method according to the protocol described by Li et al. [43], with slight modifications. Briefly, diethylenetriamine (34.3 mL) and water (5.8 mL) were mixed in a reaction flask and the mixture stirred for 5 min. Next, Cd(OAc)_2_ (0.266 g, 1 mmol) and Na_2_SeO_3_ (0.173 g, 1 mmol) were added and the mixture further stirred for 30 min at room temperature to ensure complete dissolution of precursors. Next, the solution was transferred to a teflon-lined stainless steel autoclave with 100 mL capacity and heated to 180 °C for 12 h. After cooling to room temperature, the CdSe NRs were recovered by centrifugation (4000 rpm for 15 min), washed with water (5 × 10 mL), then with ethanol (2 × 10 mL) and finally dried at 50 °C for 12 h.

### Preparation of CdSe/TiO_2_ composites

CdSe/TiO_2_ composites with a varied content of CdSe NRs (0.5, 1, 2 and 5 wt %) were prepared. For the CdSe (2 wt %)/TiO_2_ composite, commercial Aeroxide^®^ P25 TiO_2_ nanoparticles (250 mg) and CdSe NRs (5 mg) were dispersed by sonication in 5 mL water for 30 min. Next, the mixture was heated to 70 °C to evaporate water. The solid obtained was washed with water (3 × 10 mL), with ethanol (3 × 5 mL) and then calcined under air at 300 °C for 1 h to couple the CdSe NRs with TiO_2_. CdSe/TiO_2_ composites with different wt % CdSe NRs were prepared using a similar synthetic procedure.

### Photocatalytic activity measurements

The photocatalytic activity of CdSe/TiO_2_ composites was evaluated by the degradation of RhB in aqueous solution. Sylvania LuxLine FHO T5 neon tubes were used as the simulated solar light source (light intensity = 5 mW/cm^2^). A polycarbonate filter was added for experiments conducted under visible light irradiation (light intensity = 10 mW/cm^2^).

In a typical experiment, 50 mg of the CdSe/TiO_2_ composite were dispersed in 50 mL of aqueous solution of RhB (10 mg/L) in a 70 mL glass flask and the mixture was magnetically stirred in the dark under ambient conditions for 45 min to reach a thorough adsorption–desorption equilibrium. After that period, the light was turned on. Aliquots of 2 mL were taken at regular time intervals and centrifuged (4000 rpm for 2 min) to remove the CdSe/TiO_2_ photocatalyst. The relative concentration of RhB in the solution was determined by comparing its UV−visible absorption at 554 nm with that of the starting solution.

The mechanism of RhB photodegradation in aqueous solution was investigated through the use of scavengers. *t*-BuOH and *p*-benzoquinone, used as hydroxyl and superoxide scavengers, were used at concentrations of 1 and 10 mM, respectively.

### Characterization

Transmission electron microscopy (TEM) investigations were performed with a JEOL ARM 200F-Cold FEG TEM/STEM (point resolution 0.19 nm in TEM mode and 0.078 nm in STEM mode) fitted with a GIF Quatum ER. For each sample, one drop of a dispersed solution was deposited on holey carbon grids and imaged.

Scanning electron microscopy (SEM) images were prepared using a JEOL SEM JSM-6490 LV.

The crystalline phase of the powders was determined by powder X-ray diffraction (XRD) on an X'Pert MPD diffractometer (Panalytical AXS) with a goniometer radius of 240 mm, fixed divergence slit module (1/2° divergence slit, 0.04 rd Sollers slits) and an X'Celerator as a detector. The samples were placed on a silicon zero-background sample holder and the XRD patterns were recorded at room temperature using Cu Kα radiation (λ = 0.15418 nm).

XPS analysis was conducted on a Gammadata Scienta (Uppsala, Sweden) SES 200-2 spectrometer under ultra-high vacuum conditions (*P* < 10^−9^ mbar). For all peak fitting procedures the CASA XPS software (Casa Software Ltd, Teignmouth, UK, http://www.casaxps.com) was used and the areas of each component were modified according to classical Scofield sensitivity factors.

All the optical measurements were performed at room temperature (20 ± 1 °C) under ambient conditions. The absorption spectra of liquid samples were recorded on a Thermo Scientific Evolution 220 UV–visible spectrophotometer. Diffuse reflectance spectra (DRS) were recorded on a Shimadzu 2600 UV−visible spectrophotometer. BaSO_4_ powder was used as a standard for baseline measurements and spectra were recorded in a range of 250–1400 nm. Raman spectra were recorded using an Xplora spectrometer from Horiba Scientific with 532 nm wavelength incident laser light.

The initial and final total organic carbon (TOC) content was determined using a Shimadzu TOC-V_CSH_ analyzer to evaluate the degree of photomineralization.

## Results and Discussion

### Synthesis, morphological and structural characterization of CdSe/TiO_2_ composites

CdSe NRs were prepared by a solvothermal method using diethylenetriamine as structure directing agent [43]. The CdSe rods were associated to TiO_2_ nanoparticles by a heat treatment at 300 °C for 1 h to construct a heterojunction between these materials that favors the charge injection from CdSe to TiO_2_.

The microstructure of CdSe NRs and CdSe/TiO_2_ composites was first characterized by SEM (Figure S1, Supporting Information File 1). The small CdSe NRs were found to be strongly associated to each other to form larger particles with sizes varying between 1 and 4 µm. The associated energy dispersive X-ray (EDX) spectrum demonstrates that Cd and Se were the only elements detected in CdSe NRs. Due to the low content of CdSe NRs in CdSe/TiO_2_ composites, CdSe NRs could only hardly be observed by SEM. Ti, O, Cd and Se elements were detected for all composites, which indicates the successful association of CdSe NRs with TiO_2_. All CdSe/TiO_2_ composites exhibit a non-rigid texture and the pores observed should be beneficial for the diffusion of molecules and thus for an enhanced photocatalytic activity.

**Figure 1:**
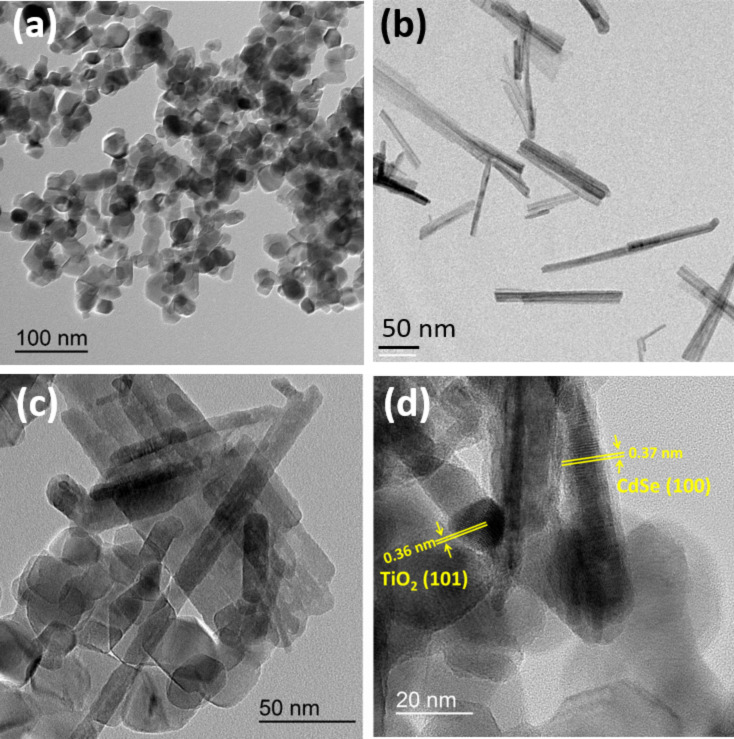
TEM images of (a) TiO_2_, (b) CdSe nanorods and (c) the CdSe (2 wt %)/TiO_2_ composite. (d) HR-TEM image of CdSe nanorods associated to TiO_2_ nanoparticles.

The morphology of the particles was further characterized by TEM. Commercial P25 TiO_2_ nanoparticles (Aeroxide^®^ P25) are of spherical/ellipsoidal shape and their average diameter is ≈23 ± 6 nm (Figure 1a). CdSe NRs produced after the solvothermal synthesis have an average length and an average diameter of 120 and 12 nm, respectively (Figure 1b). Some nanowires with lengths up to 300 nm could also be detected. A typical TEM image of the CdSe (2 wt %)/TiO_2_ composite is given in Figure 1c and demonstrates the coexistence and the association of CdSe NRs and TiO_2_ particles. This could be further confirmed by HR-TEM (Figure 1d). The measured lattice spacing was 0.36 and 0.37 nm, corresponding to the (101) plane of anatase TiO_2_ and to the (100) plane of wurtzite CdSe, respectively.

**Figure 2:**
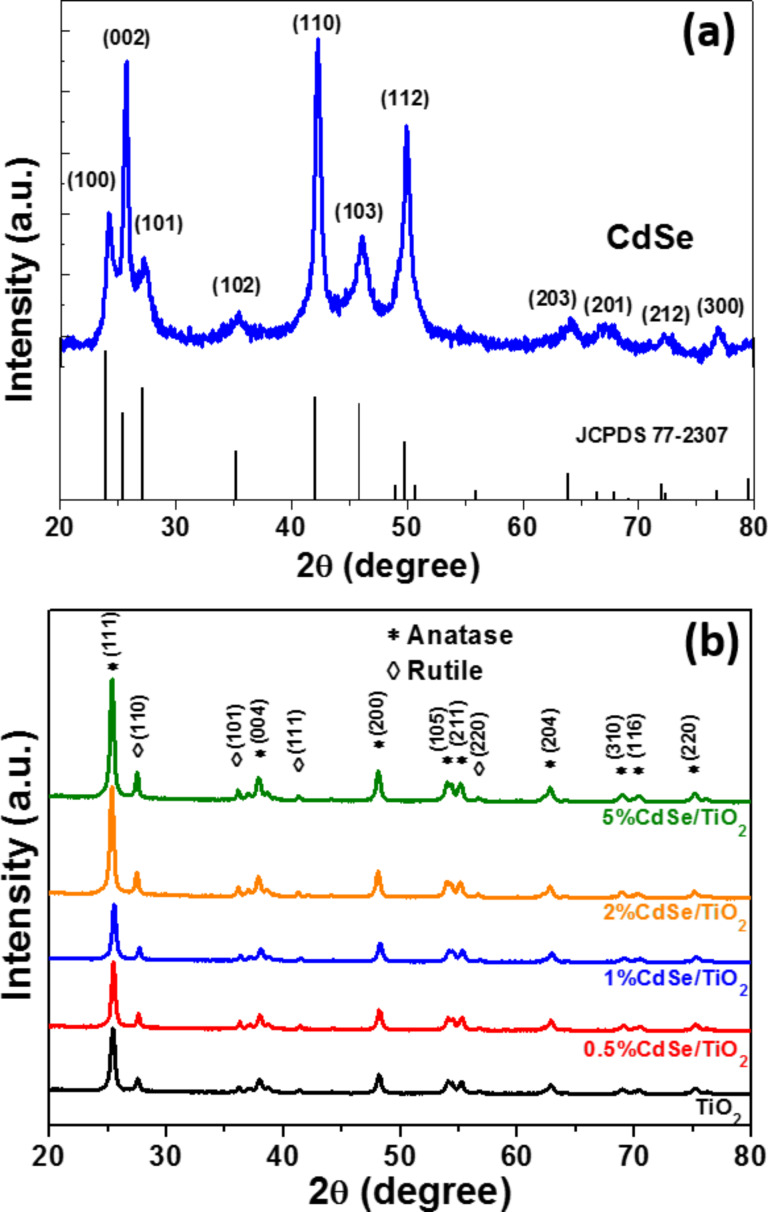
X-ray diffraction patterns of (a) CdSe and (b) TiO_2_ and CdSe/TiO_2_ composites with varying CdSe wt % from 0.5 to 5 wt %.

XRD analysis of CdSe, TiO_2_ and CdSe-NR-sensitized TiO_2_ is presented in Figure 2. For pure CdSe rods, all peaks could be indexed to the hexagonal wurtzite structure (JCPDS No 77-2307), which is in accordance with the previously described HR-TEM results (Figure 2a). For TiO_2_, the diffraction peaks at 2θ = 25.41, 37.98, 48.18, 54.12, 55.24, 62.93, 69.03, 70.42 and 75.17° can be indexed to the (111), (004), (200) (105), (211), (204), (310), (116) and (220) crystal planes of anatase TiO_2_ (JCPDS No 21-1272), while those located at 2θ = 27.55, 36.23, 41.34 and 56.72° belong to rutile TiO_2_ (JCPDS No 21-1276) (Figure 2b). The anatase and rutile phases of TiO_2_ exhibit a higher catalytic activity than the brookite phase [44]. The anatase/rutile ratio was not affected by the heating at 300 °C (the phase transition occurs at 500 °C). The position of TiO_2_ diffraction peaks was not changed after association with CdSe, indicating that CdSe NRs do not affect the lattice structure of TiO_2_. The diffraction peaks of CdSe NRs could not be observed in all CdSe/TiO_2_ composites due to the low content of CdSe (from 0.5 to 5 wt %) and their dispersion in TiO_2_ particles.

**Figure 3:**
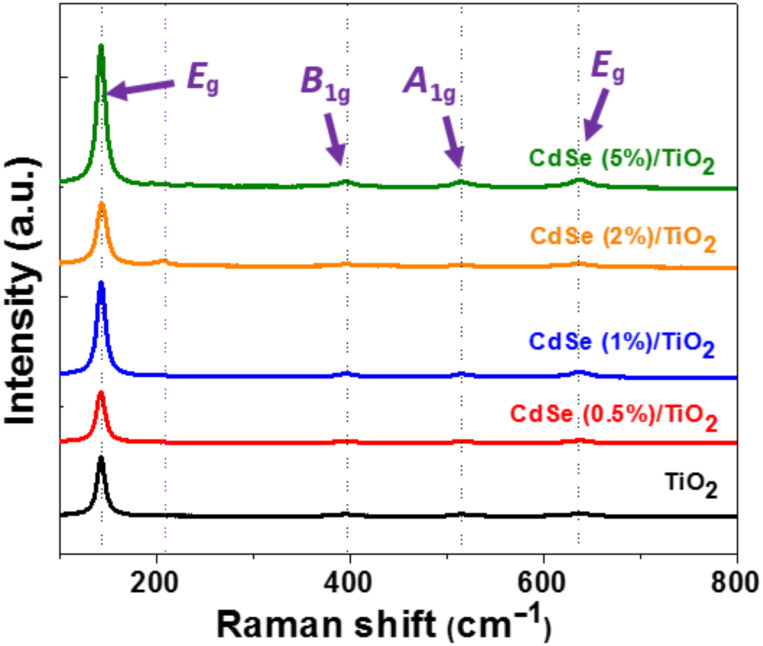
Raman spectra of TiO_2_ and CdSe/TiO_2_ composites.

Raman spectroscopy was further used to confirm the structures of CdSe/TiO_2_ composites. Two peaks located at 206 and 414 cm^−1^ corresponding to the longitudinal optical (LO) phonon and its overtone (2LO) can be observed for CdSe NRs (Figure S2, Supporting Information File 1) [45]. For TiO_2_, the *E*_g_, *B*_1g_, *A*_1g_ and *E*_g_ peaks located at 143, 397, 514 and 636 cm^−1^, respectively, are typical of the TiO_2_ anatase phase [46] (Figure 3). Due to the weak content in CdSe NRs, the LO signal of CdSe could only be detected for composites containing 2 and 5 wt % CdSe NRs. However, Raman analysis indicates that CdSe NRs are well-associated to TiO_2_ particles.

**Figure 4:**
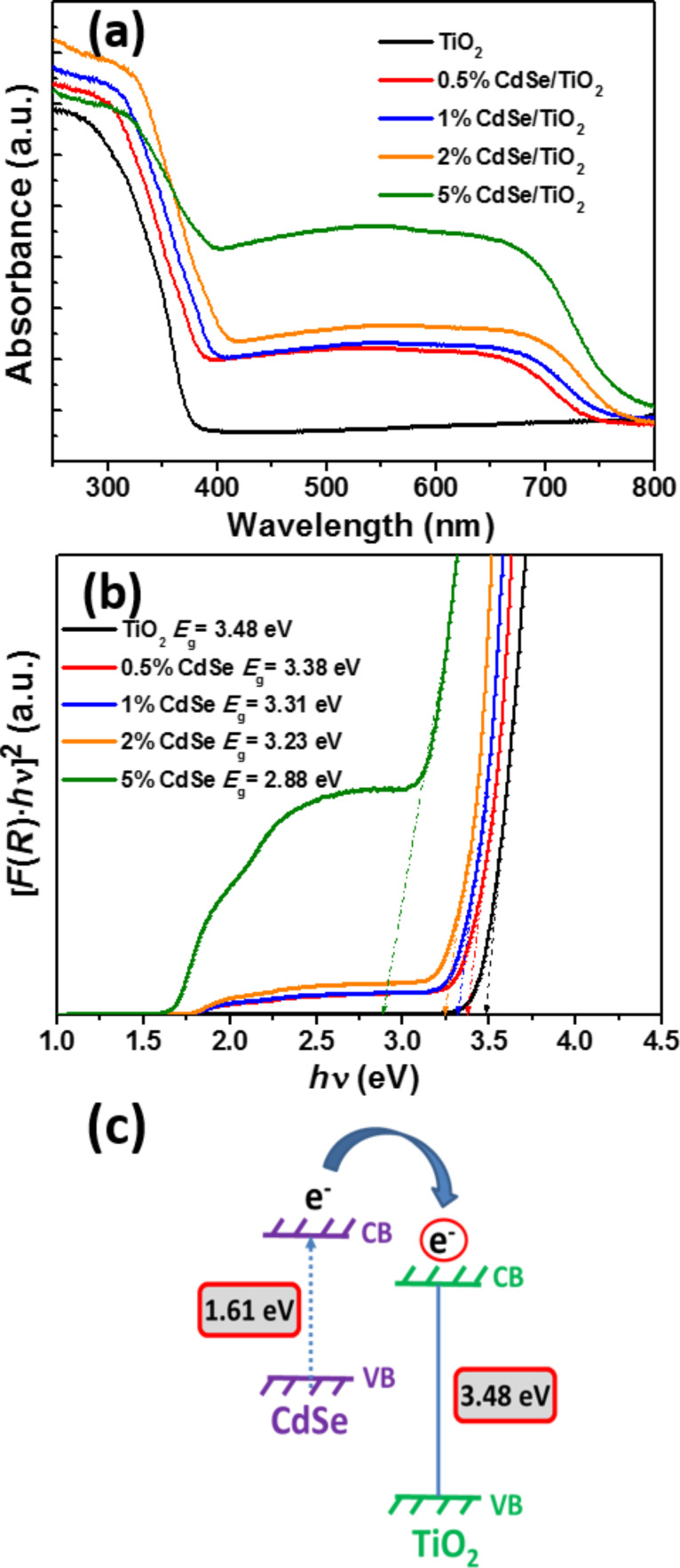
(a) UV–visible absorption spectra of TiO_2_ and CdSe/TiO_2_ composites, (b) plots of transformed Kubelka–Munk functions [*F*(*R*)·*hν*]^2^ vs *hν* for TiO_2_ and CdSe/TiO_2_ composites, and (c) energy level diagram for CdSe NRs/TiO_2_ composites.

The UV–visible absorption spectra of TiO_2_ and CdSe/TiO_2_ composites are shown in Figure 4a. The bandgap energies of TiO_2_, CdSe and CdSe/TiO_2_ composites were determined by plotting [*F*(*R*)*h*ν]^2^ vs photon energy and extrapolating the plots at [*F*(*R*)*hν*]^2^ = 0 (*F*(*R*) is the Kubelka–Munk function, *h* is the Planck constant and *ν* is the frequency) (Figure 4b). No optical response in the visible region could be detected for TiO_2_ due to its wide bandgap (3.48 eV). As can be seen, the absorption increases with the increase of CdSe NRs in the composite from 400 to ≈725 nm, which corresponds to the energy bandgap of CdSe NRs (≈1.61 eV) – a value slightly lower than the standard bandgap of CdSe (1.78 eV) [47]. This enhanced visible light absorption originates from the excitation of CdSe NRs and indicates a decrease of the bandgap with increasing amount of CdSe (the bandgap of CdSe/TiO_2_ composites was found to decrease from 3.38 eV for 0.5 wt % CdSe to 2.88 eV for 5 wt % CdSe) (Figure 4b). Because the conduction band edge of CdSe NRs is higher than that of TiO_2_, the results shown in Figure 4 demonstrate that the excited electrons of CdSe NRs can be transferred to TiO_2_ nanoparticles due to the heterojunction between these materials (Figure 4c). This should contribute to enhance the photocatalytic activity of CdSe/TiO_2_ composites under visible light irradiation (vide infra).

**Figure 5:**
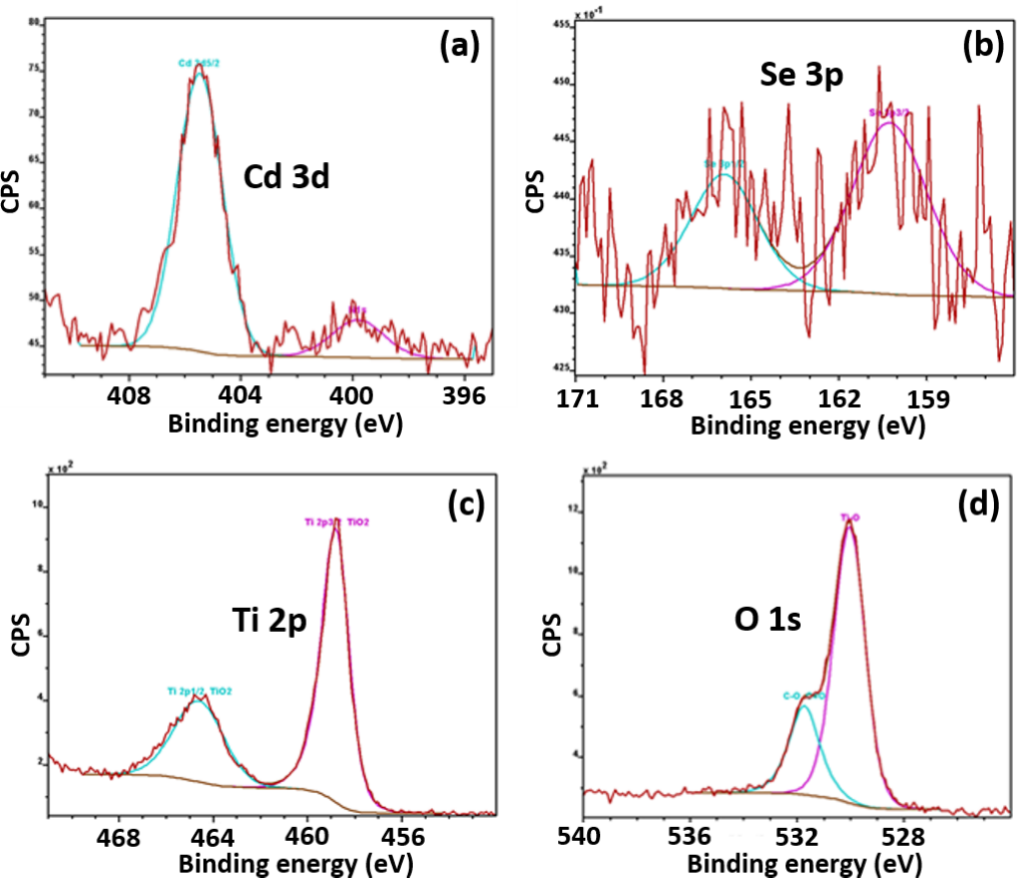
High-resolution XPS spectra of the CdSe (2 wt %)/TiO_2_ composite: (a) Cd 3d, where Cd 3d_5/2_ (blue) at 405.46 eV comprised 45.18% of the area of the spectra and N 1s (pink) at 399.81 eV comprised 54.82% of the area of the spectra; (b) Se 3p, where Se 3p_1/2_ (blue) at 165.91 eV comprised 50.09% of the area of the spectra and Se 3p_3/2_ (pink) at 160.26 eV comprised 49.91% of the area of the spectra; (c) Ti 2p, where Ti 2p_1/2_ (blue) at 464.56 eV comprised 44.72% of the area of the spectra and Ti 2p_3/2_ (pink) at 458.74 eV comprised 55.28% of the area of the spectra and (d) O 1s, where C–O C=O (blue) at 531.72 eV comprised 24.81% of the area of the spectra and Ti–O (pink) at 530.00 eV comprised 75.19% of the area of the spectra. CPS = counts per second.

The chemical state of the elements composing the CdSe (2 wt %)/TiO_2_ composite was studied by XPS. The high-resolution spectra of the elements Cd 3d_5/2_, Se 3p_3/2,_ Cd 3p_1/2_, Ti 2p and O 1s in the CdSe (2 wt %)/TiO_2_ composite are provided in Figure 5. The signals of Ti 2p_3/2_, Ti 2p_1/2_, O 1s (lattice oxygen atoms), and O 1s (surface hydroxyl groups) appear at 458.74, 464.56, 530.0, and 531.76 eV, respectively, which are values in good accordance with those measured for pure TiO_2_ [48]. The signals of Cd 3d_5/2_ and Se 3p_3/2_ and Se 3p_1/2_ can be observed at 405.46, 160.26 and 165.91 eV, respectively. These values are typical for CdSe [49] and further confirm that CdSe NRs are associated to TiO_2_. Both for CdSe and TiO_2_, no significant shift in binding energy was observed after building the heterojunction between these materials (see Supporting Information File 1, Figure S3 for the high-resolution XPS spectra of Cd 3d_5/2_ and Se 3p of CdSe NRs). Noteworthy is also that the surface of CdSe NRs was not oxidized into CdO during the calcination step. The signals of Cd 3d_5/2_ at ≈403.2 eV for CdO [50] and Se 3p_3/2_ at 165.1 eV for SeO_2_ [51] were not detected in the XPS spectra of the CdSe (2 wt %)/TiO_2_ composite.

### Solar light photocatalytic activity of CdSe/TiO_2_ composites

**Figure 6:**
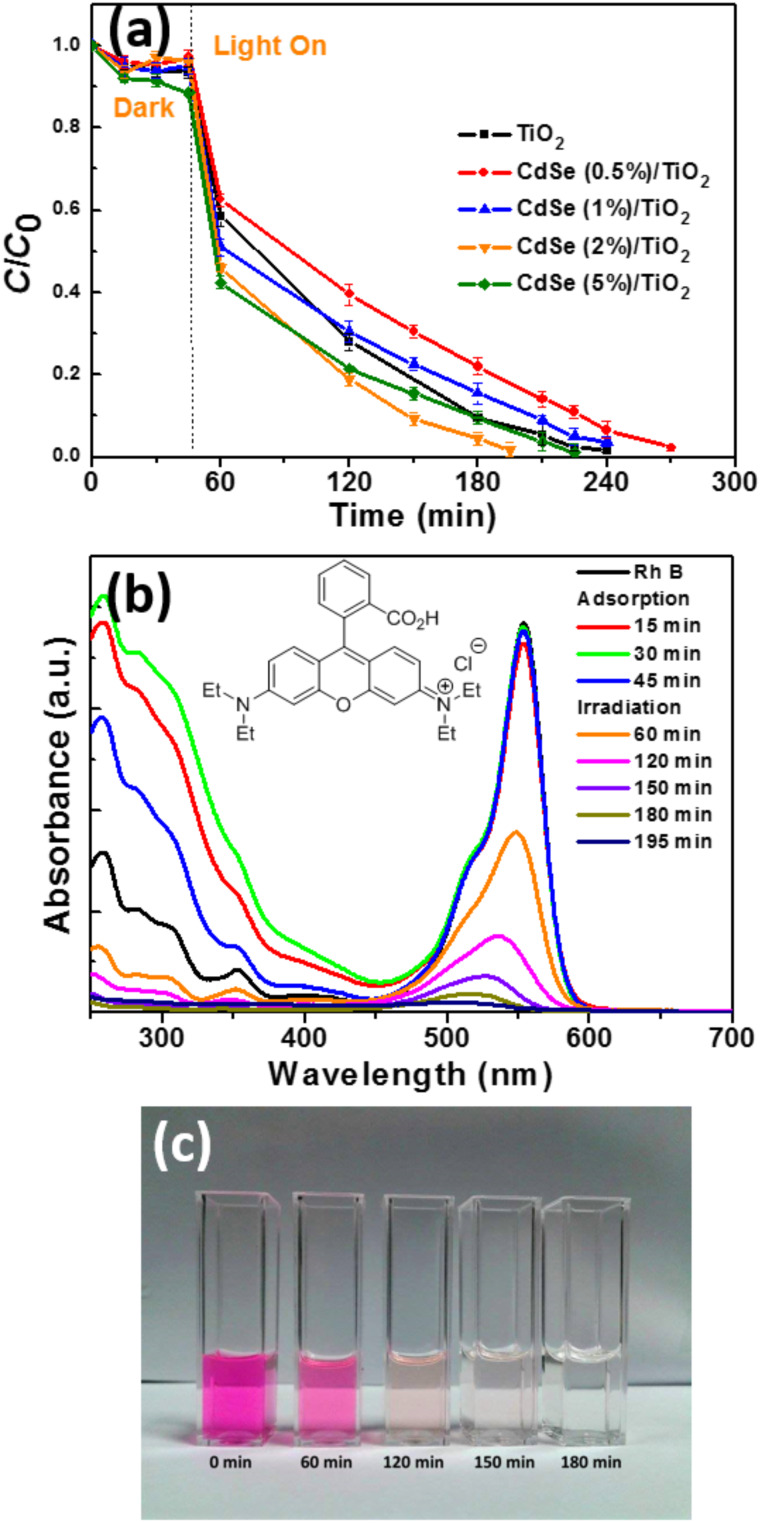
(a) Photocatalytic activity of TiO_2_ and CdSe/TiO_2_ composites for the degradation of RhB under simulated solar light irradiation, (b) UV–visible absorption spectra of RhB during the photodegradation using the CdSe (2 wt %)/TiO_2_ composite (the inset shows the chemical structure of RhB), and (c) a digital photograph of the RhB solution over the course of the photocatalytic degradation.

The photocatalytic decomposition of RhB at pH 7 over TiO_2_ nanoparticles and CdSe/TiO_2_ composites under simulated solar light irradiation was first evaluated (light intensity: 5 mW/cm^2^). Before illumination, the adsorption/desorption equilibrium was established for 45 min. In the absence of the photocatalyst, the photodegradation of RhB was found to be negligible. As shown in Figure 6a, the highest photocatalytic activity was reached for the CdSe (2 wt %)/TiO_2_ material which fully bleached the dye in 150 min. Thereafter, the activity decreases when increasing the amount of CdSe NRs associated to TiO_2_. Because the photocatalytic activity originates from the spatial separation of photogenerated electrons and holes, it is likely that CdSe NRs are not properly associated to TiO_2_ and do not contribute to the photocatalytic process.

Total organic carbon (TOC) measurements showed that the RhB solution was discolored during photocatalytic experiments (Figure 6c) and the dye was also partially decomposed (the TOC value decreased from 6.9 ± 0.3 to 2.4 ± 0.2 mg/L after the 150 min of irradiation).

The temporal evolution of the UV–vis absorption spectra is displayed in Figure 6b. The intensity of the RhB absorption from 250 to 600 nm decreases over time. A blue shift (from 553 to 503 nm) of the main absorption peak is also observed, which is indicative of stepwise *N*-de-ethylation of the dye during the photocatalytic process [52]. The plots of ln(*C*_0_/*C*) of RhB vs irradiation time indicate that the photodegradation of RhB over CdSe/TiO_2_ composites follows a pseudo-first-order kinetic model (*C*_0_ is the initial concentration of RhB and *C* is the concentration of the dye at time *t*). The rate constants *k* determined from the slopes of ln(*C*_0_/*C*) vs time were found to be 0.017, 0.012, 0.013, 0.019, and 0.014 min^−1^ for TiO_2_ and CdSe/TiO_2_ composites loaded with 0.5, 1, 2, and 5 wt % CdSe, respectively (Figure S4, Supporting Information File 1).

### Influence of the photocatalyst dosage and rhodamine B concentration

**Figure 7:**
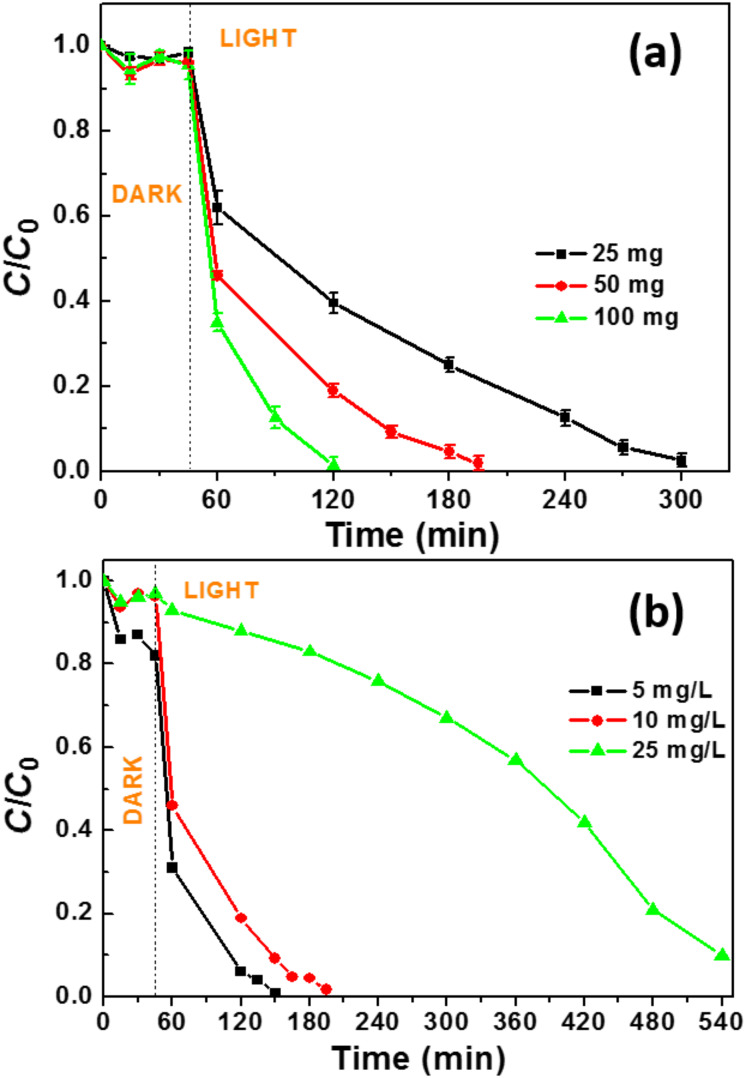
(a) Effect of the catalyst concentration on the photodegradation efficiency (25, 50 or 100 mg of photocatalyst were dispersed in 50 mL of a 10 mg/L RhB solution). (b) Influence of RhB concentration on the photocatalytic efficiency (50 mg of photocatalyst and 50 mL of the dye solution were used).

The photodegradation efficiency is generally affected by the amount of catalyst used and by the concentration of the pollutant. First, we investigated the influence of the catalyst concentration (25, 50 or 100 mg in 50 mL of RhB solution) (Figure 7a). Using 25 mg of the CdSe (2 wt %)/TiO_2_ photocatalyst, the dye decomposition required 255 min while the degradation could be achieved within 75 min using 100 mg of photocatalyst. As expected, the photocatalytic degradation rates increased with the catalyst dosage, which is linked to the concentration of adsorption sites and photocatalytically active sites (*k* = 0.010, 0.019 and 0.034 min^−1^ for reactions conducted with 25, 50 and 100 mg of photocatalyst, respectively).

Next, the concentration of the RhB solution was varied from 5, 10 to 25 mg/L while the amount of catalyst was fixed at 50 mg (Figure 7b). The photodegradation rate decreases with increasing RhB concentration (*k* = 0.028, 0.019 and 0.002 min^−1^ for RhB concentrations of 5, 10 and 25 mg/L, respectively). This originates (i) from the high amount of dye and/or the photodegradation intermediates adsorbed at the photocatalyst surface and (ii) from the decrease of the light penetration in the reactor due to the high absorption of RhB and thus to the decreased amount of reactive oxygen species produced.

### Influence of the pH of the rhodamine B solution

**Figure 8:**
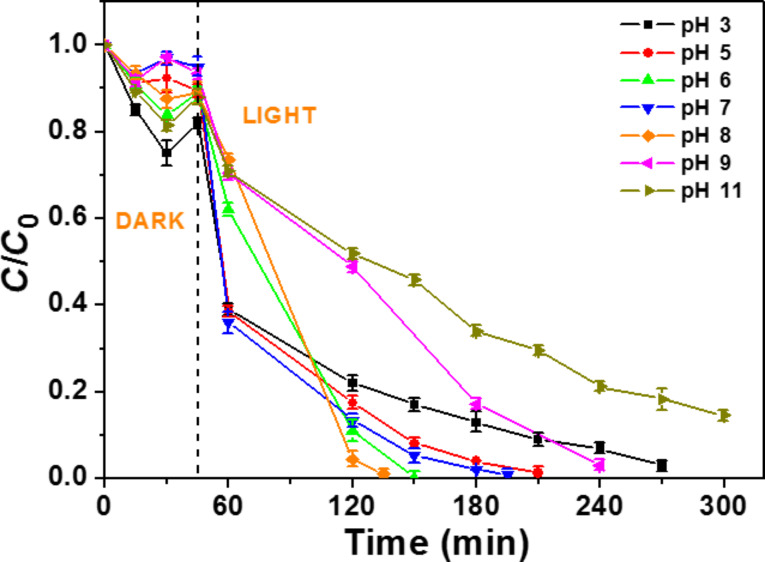
Influence of pH on the degradation of rhodamine B using the CdSe (2 wt %)/TiO_2_ photocatalyst.

The pH of the aqueous solution may play a crucial role on the kinetic of the photodegradation since it influences the surface charge of the catalyst and the structure of the dye in solution. Figure 8 shows that the CdSe (2 wt %)/TiO_2_ photocatalyst exhibits the highest activity at pH values varying from 5 to 8 (*k* values are varying from 0.017 to 0.031 min^−1^). At pH 3, the photodegradation kinetic is reduced (k = 0.011 min^-1^) but the highest inhibition was observed in basic media (*k* = 0.009 and 0.006 min^−1^ at pH 9 and 11, respectively). The point of zero charge of the TiO_2_ nanoparticles is at ≈6.5 [53] and the p*K*_a_ of the acid function of RhB is 3.7. At pH ≥ 9, the weak photocatalytic activity probably originates from the strong repellency between the carboxylate function of RhB and the negatively charged catalyst. At pH 3, the positively charged RhB exhibits only a modest affinity for the positively charged catalyst. At pH values ranging from 5 to 8, RhB may associate to the photocatalyst either via interaction involving the carboxylate function or the positively charged diethylamino groups.

### Visible light photocatalytic activity of the CdSe (2 wt %)/TiO_2_ composite

**Figure 9:**
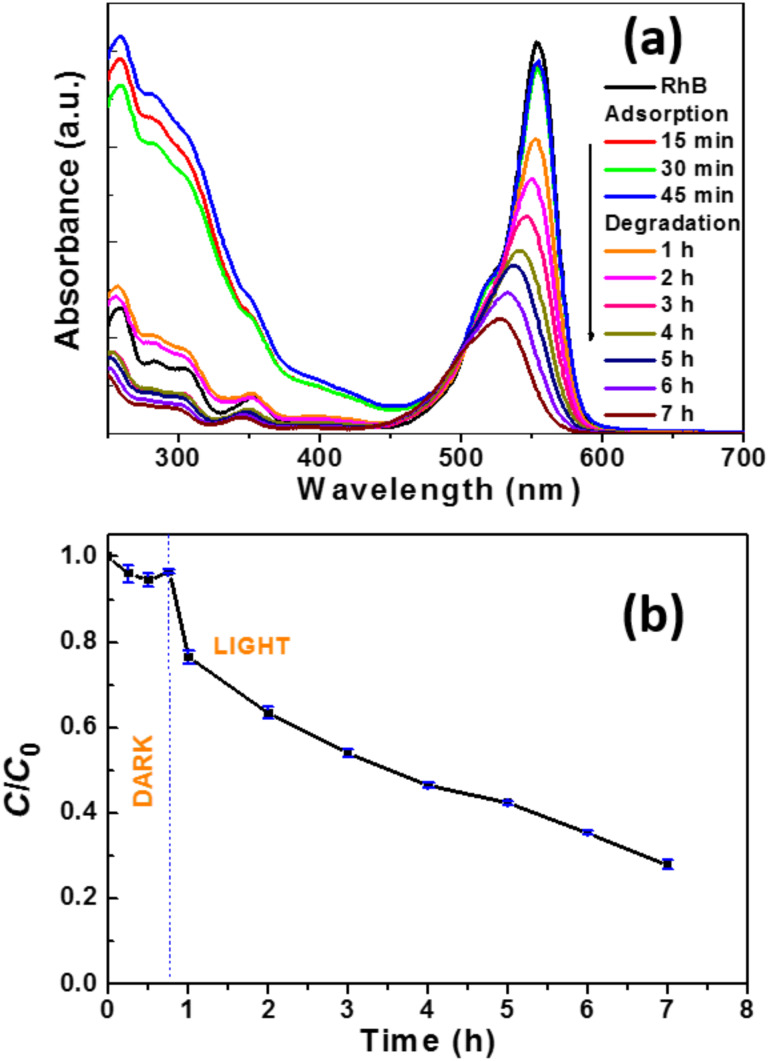
(a) UV–visible spectral evolution of rhodamine B as a function of irradiation time using the CdSe (2 wt %)/TiO_2_ catalyst under visible light irradiation (light intensity: 10 mW/cm^2^) and (b) evolution of *C*/*C*_0_ vs irradiation time.

The CdSe (2 wt %)/TiO_2_ composite is also photocatalytically active when the incident light wavelength is greater than 400 nm. The spectral evolution of RhB and the degradation profile are shown in Figure 9. As can be seen, the photodegradation is slower than that initiated by simulated solar light and ≈70% of RhB is bleached after 6.25 h of irradiation. A similar hypsochromic shift of the absorption band at 553 nm to that observed under simulated sunlight irradiation was observed, indicating that photooxidative *N*-de-ethylation of RhB occurred in the early stages of its photodegradation. Finally, although the operating conditions and the structure of the pollutant were different from those used in this study, the CdSe NR/TiO_2_ nanoparticle photocatalyst favorably compares with catalysts engineered from CdSe quantum dots and TiO_2_ nanoparticles or nanotubes [9,11–12,22,26,31,33–34].

### Reusability of the CdSe (2 wt %)/TiO_2_ photocatalyst

**Figure 10:**
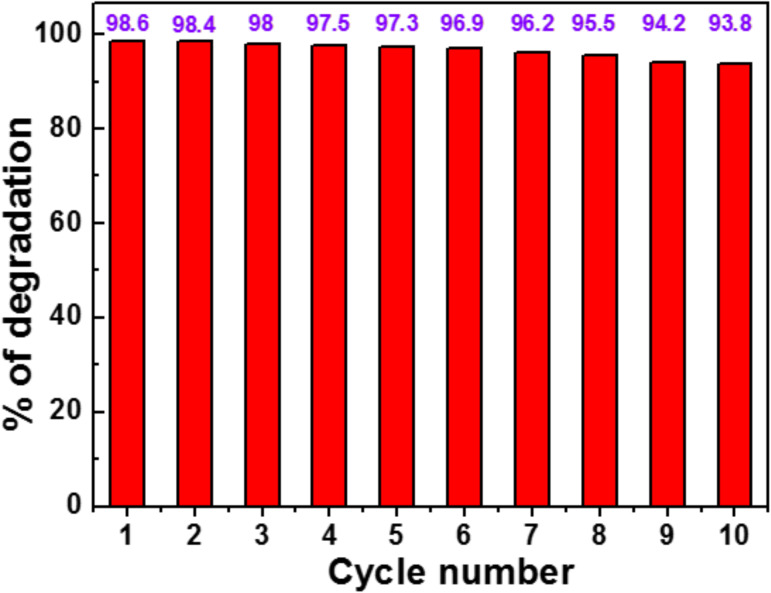
Recycling of the CdSe (2 wt %)/TiO_2_ catalyst in the degradation of RhB under simulated solar light irradiation.

The CdSe (2 wt %)/TiO_2_ photocatalyst was subjected to reuse under simulated solar light irradiation. After the first run, the photocatalyst was recovered by centrifugation (4000 rpm for 15 min) and reused without any treatment. As can be seen from Figure 10, no marked decrease in photocatalytic activity was observed after ten reuses, indicating that byproducts originating from RhB decomposition do not block the active sites on the photocatalyst surface and that the photocatalyst is not photocorroded. Furthermore, XRD patterns of the CdSe (2 wt %)/TiO_2_ composite before and after ten reuses and the SEM analysis clearly demonstrate that the photocatalyst is stable during the reaction (Supporting Information File 1, Figure S5). The stability of the CdSe/TiO_2_ catalyst is of high importance for real photocatalytic applications.

### Photodegradation mechanism

**Figure 11:**
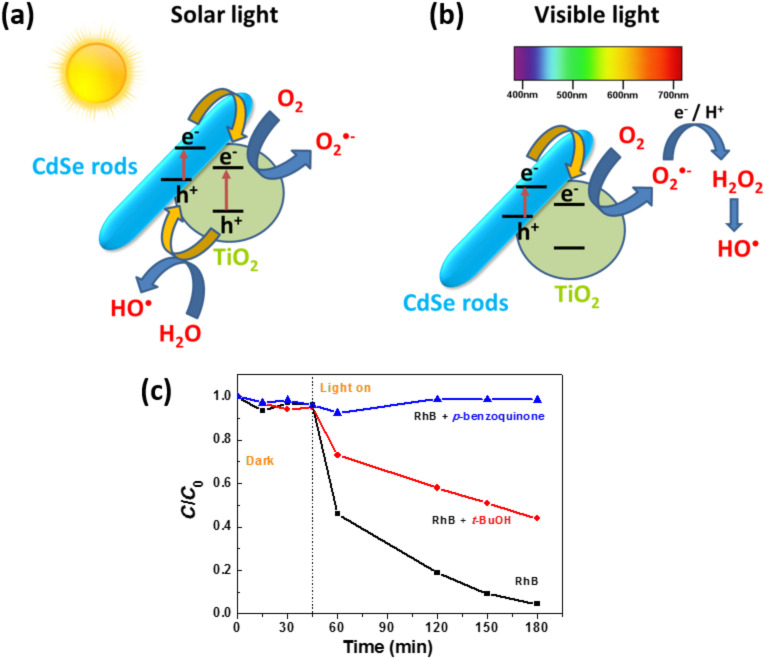
Schematic of the charge separation in the CdSe/TiO_2_ photocatalyst under (a) solar light and (b) visible light irradiation. (c) The influence of O_2_^•−^ and ^•^OH scavengers on the photocatalytic degradation of rhodamine B under visible light irradiation (*p*-benzoquinone and *t*-BuOH were used at concentrations of 1 mM and 10 mM, respectively).

Under simulated solar light irradiation, electron (e^−^)/hole (h^+^) pairs are generated both in CdSe NRs and TiO_2_ nanoparticles. The holes in the valence band (VB) of TiO_2_ are transferred to the VB of CdSe NRs while electrons are transferred from the conduction band (CB) of CdSe NRs to the CB of TiO_2_ (Figure 11a). Under visible light illumination, only CdSe can be activated. A CdSe NR electron is promoted from the VB to the CB, leaving a hole in the VB. Then, the electron is transferred to TiO_2_ (Figure 11b). All these transfer processes are thermodynamically favorable since both the CB and VB of CdSe NRs lie above that of TiO_2_. These charge separations may effectively reduce the probability of recombination and increase the lifetime of charge carriers (e^−^ and h^+^).

Under visible light irradiation, holes in the VB of CdSe may directly oxidize the dye. Meanwhile, the electrons accumulated in TiO_2_ can be trapped by dissolved oxygen molecules and generate superoxide O_2_^•−^ radicals which are strong oxidants able to decompose organic substances. These O_2_^•−^ radicals may also react with an electron and protons to form hydrogen peroxide which is further decomposed into hydroxyl ^•^OH radicals able to oxidize RhB.

To estimate which of these reactive oxygen species plays a key role in the photodegradation of RhB under visible light irradiation, experiments were carried out by adding *t*-BuOH and *p*-benzoquinone, used as ^•^OH and O_2_^•−^ radicals scavengers, respectively (Figure 11c). As can be seen, RhB could not be photodegraded in the presence of *p*-benzoquinone, even when used at a low concentration (1 mM), indicating that O_2_^•−^ radicals play a key role in the photodegradation. In the presence of *t*-BuOH (10 mM), the reaction rate was markedly decreased (*k* = 0.004 min^−1^ vs 0.012 min^−1^ in the absence of *t*-BuOH) but RhB can still be photodegraded. On the basis of these results, the photodegradation catalyzed by CdSe/TiO_2_ composites under visible light irradiation occurs mainly via O_2_^•−^ radicals and to a lesser extend via ^•^OH radicals. It is therefore likely that the heterojunction between CdSe and TiO_2_ favors the transfer of electrons in the CB of TiO_2_ followed by the production of O_2_^•−^ radicals (Figure 11b).

## Conclusion

In this study, CdSe NRs with an average length of ≈120 nm were successfully associated to TiO_2_ particles to form heterostructured CdSe/TiO_2_ photocatalysts. Due to their structure, the CdSe/TiO_2_ composites exhibit full visible and near-infrared light absorption and improved photocatalytic activity via charge separation between CdSe and TiO_2_ materials. The CdSe (2 wt %)/TiO_2_ composite was demonstrated to be the more efficient for the degradation of rhodamine B both under simulated sunlight or visible light irradiation. This facile approach for the preparation of a CdSe NRs/TiO_2_ photocatalyst, along with the high stability and low sensitivity to pH changes, demonstrates the high potential of this material for practical photocatalytic applications. The results described in this work stimulate the idea that CdSe/TiO_2_ composites may be of high potential for other applications, such as CdSe-sensitized TiO_2_ films for solar cells.

## Supporting Information

File 1 Additional figures.
