# Supplementary material for: CdSe nanorod/TiO2 nanoparticle heterojunctions with enhanced solar- and visible-light photocatalytic activity
Source: Beilstein J Nanotechnol. 2017 Dec 19;8:2741–52. doi: 10.3762/bjnano.8.273 (PMC5753051; doi:10.3762/bjnano.8.273)
Supplement: File 1 — Additional figures. [file Beilstein_J_Nanotechnol-08-2741-s001.pdf]

Supporting Information

for

CdSe nanorod/TiO_2 nanoparticle heterojunctions with enhanced solar- and visible-light photocatalytic activity

Fakher Laatar[1], Hatem Moussa[2], Halima Alem[3], Lavinia Balan[4], Emilien Girot[2], Ghouti Medjahdi[3], Hatem Ezzaouia[1] and Raphaël Schneider[*2]

Address: [1]Laboratory of Semiconductors, Nanostructures and Advanced Technology (LSNTA), Center for Research and Technology Energy, Tourist Route Soliman, BP 95, 2050 Hammam-Lif, Tunisia, [2]Laboratoire Réactions et Génie des Procédés (LRGP), UMR 7274 CNRS Université de Lorraine, 1 rue Grandville 54001 Nancy, France, [3]Institut Jean Lamour (IJL), UMR 7198 CNRS Université de Lorraine, BP 70239, 54506 Vandoeuvre-lès-Nancy Cedex, France, and [4]Institut de Science des Matériaux de Mulhouse (IS2M), CNRS UMR 7361, 15 rue Jean Starcky, 68093 Mulhouse, France

Email: Raphaël Schneider - raphael.schneider@univ-lorraine.fr

* Corresponding author

Additional figures

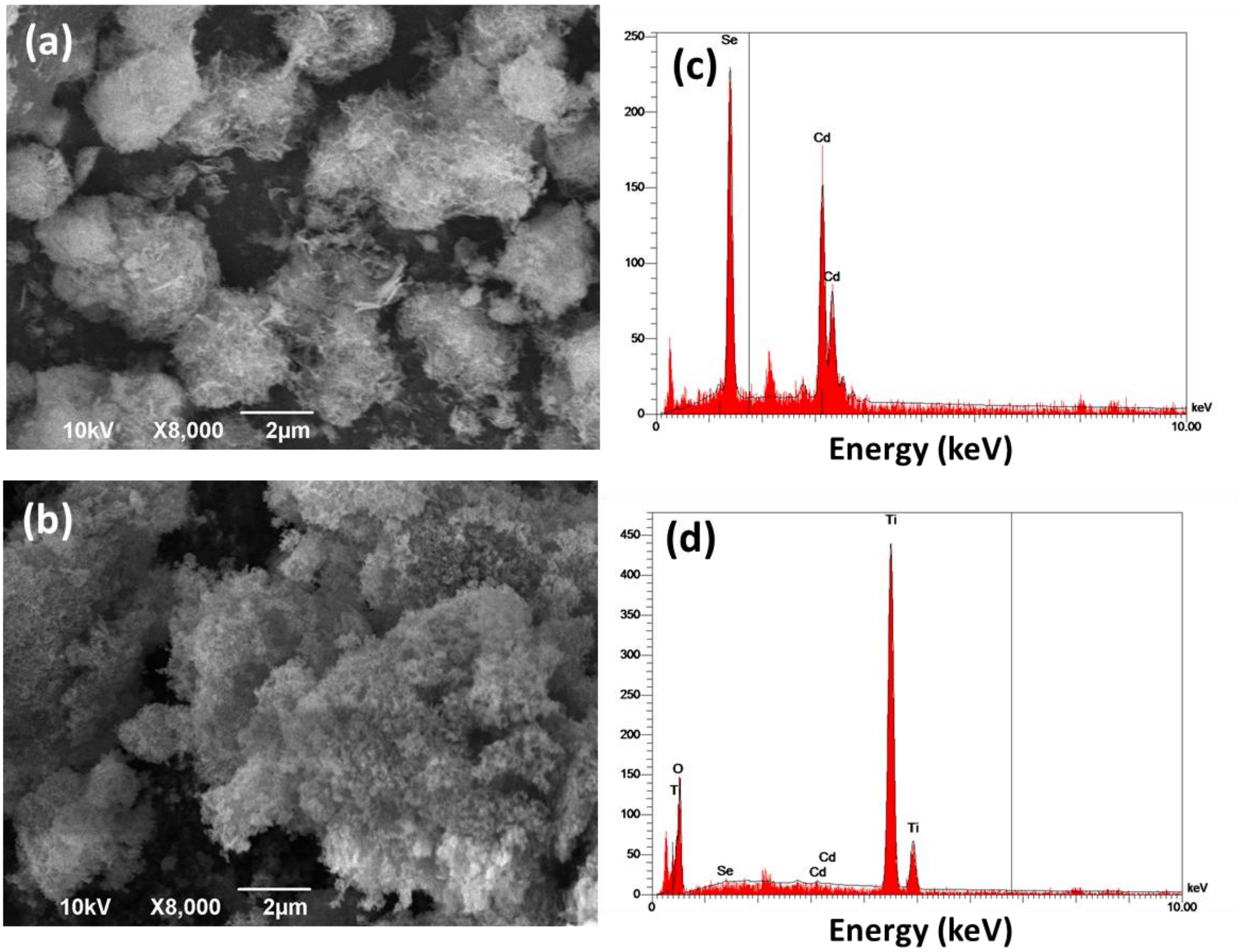

Figure S1: SEM images of (a) CdSe NRs and (b) CdSe (2%)/TiO_2 particles. (c) and (d) are the associated EDX analyses.

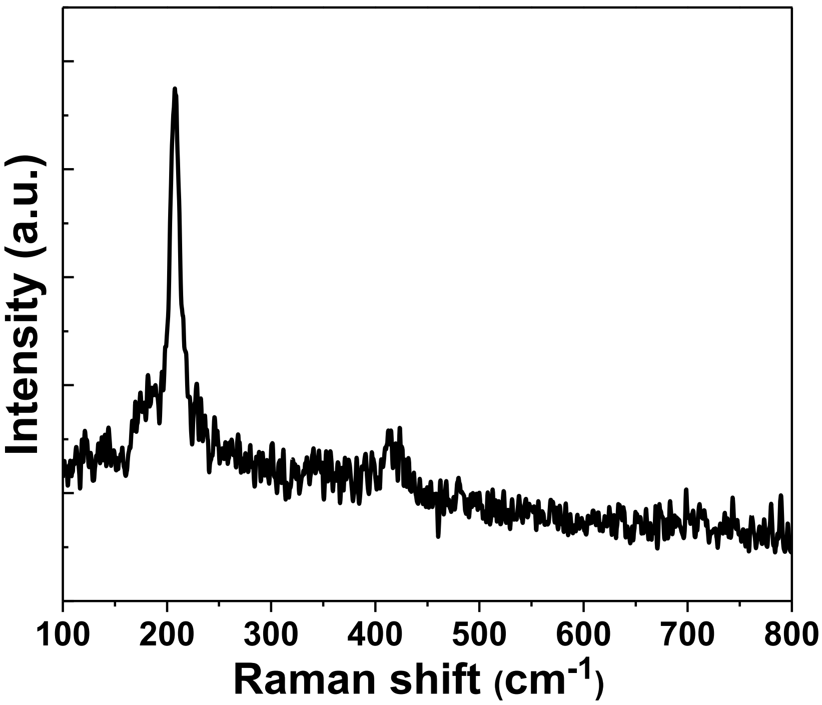

Figure S2: Raman spectrum of CdSe NRs.

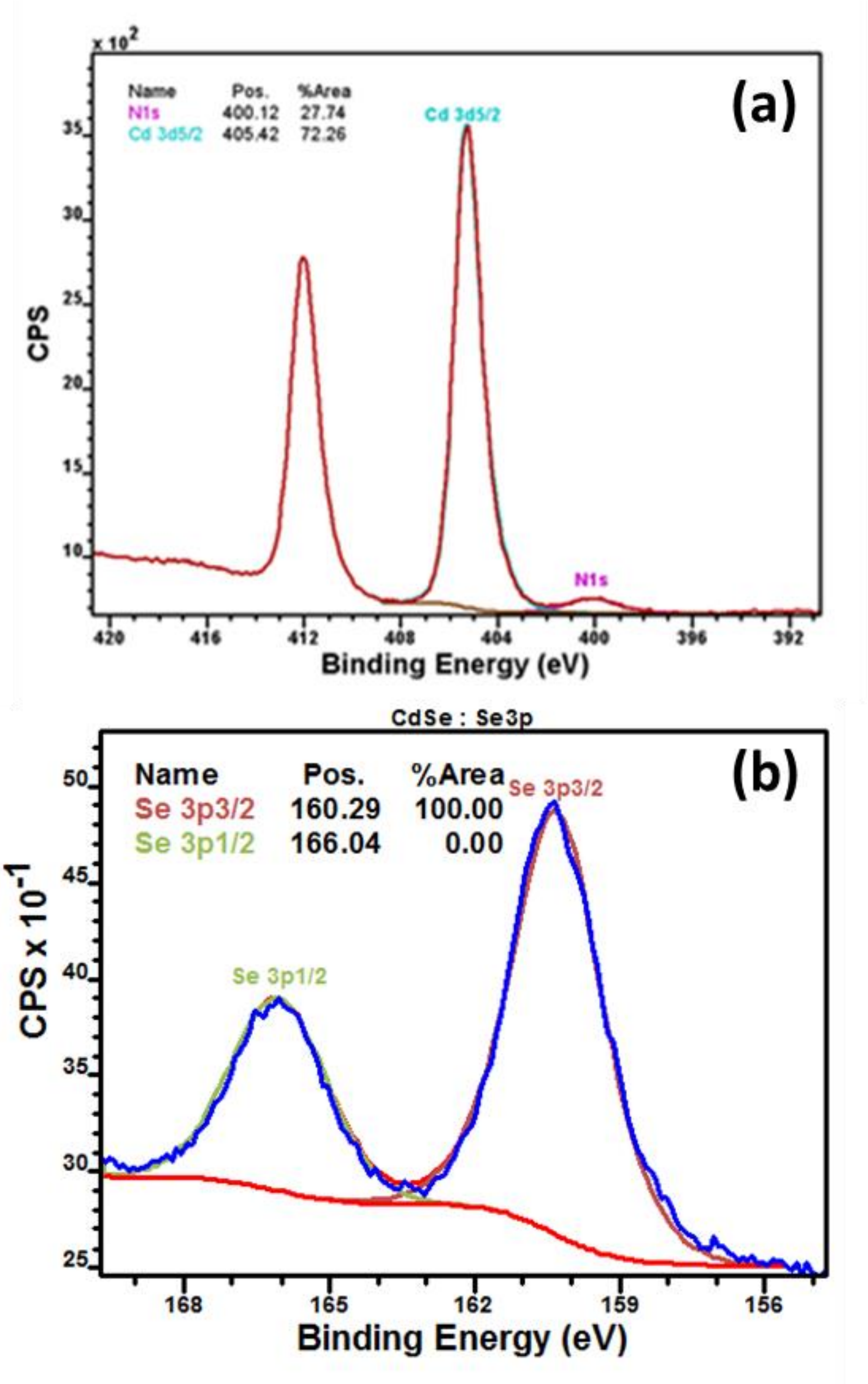

Figure S3: High resolution XPS spectra of (a) Cd $3d_{5/2}$, (b) Se 3p recorded for CdSe NRs.

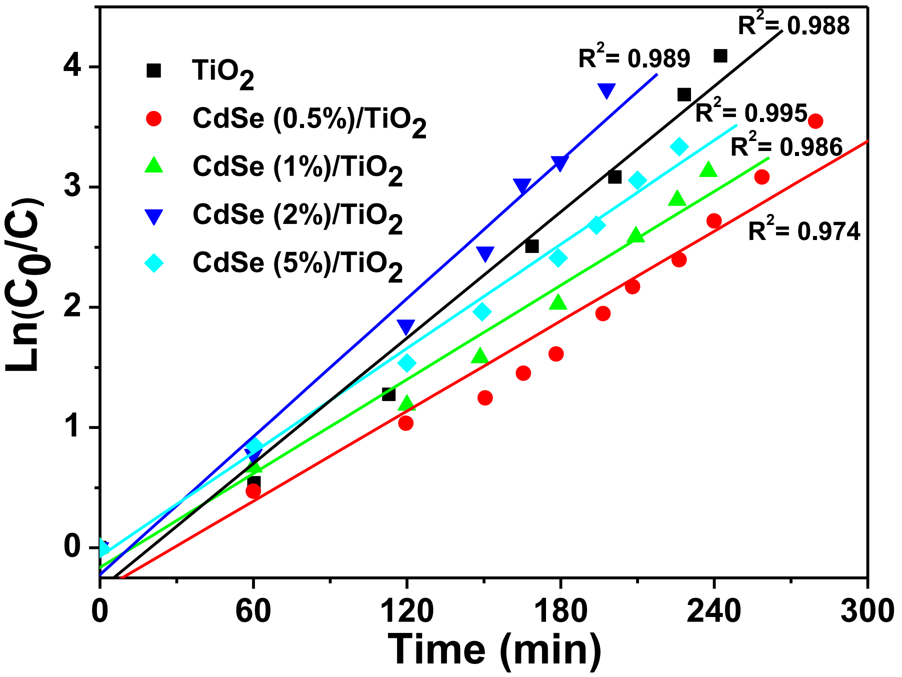

Figure S4: Pseudo first-order kinetics of RhB degradation using TiO_2 and $CdSe/TiO_2$ composites.

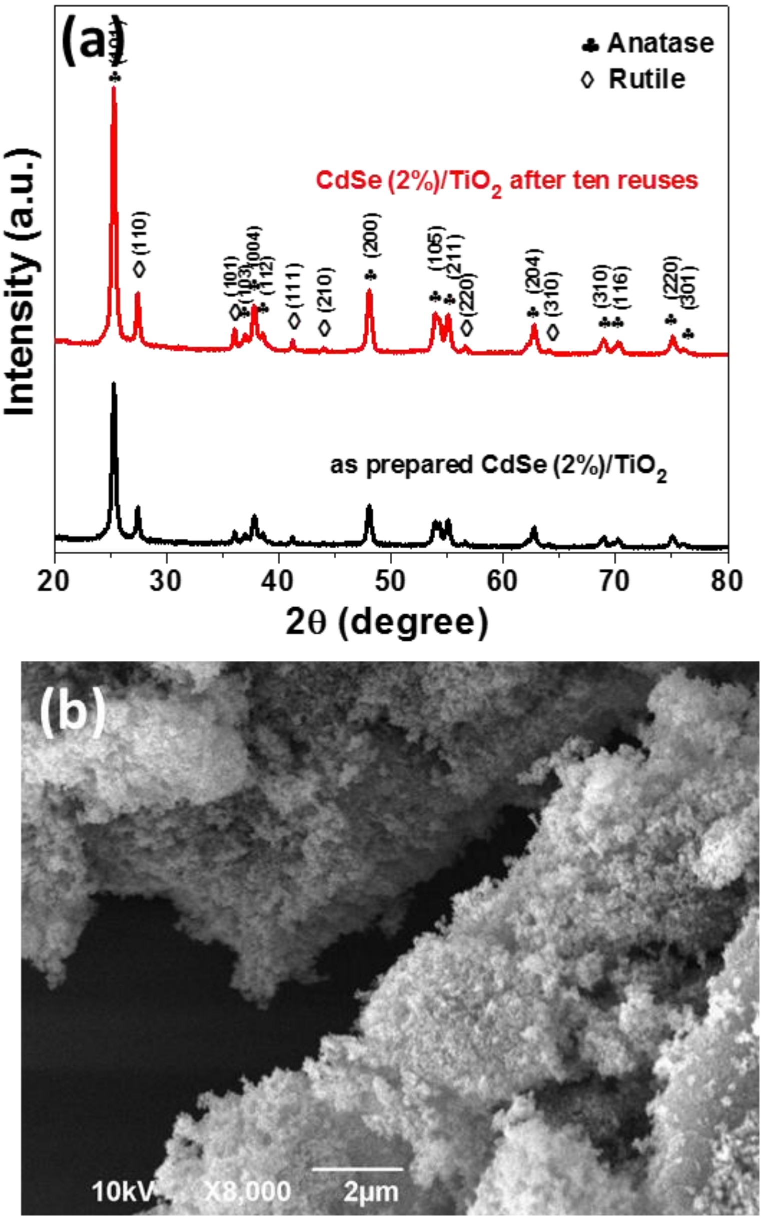

Figure S5: (a) XRD patterns of the CdSe (2%)/TiO_2 photocatalyst after synthesis and after ten reuses. (b) SEM image of the CdSe (2%)/TiO_2 photocatalyst after ten reuses.
